# Exercise interventions for nonspecific low back pain: a bibliometric analysis of global research from 2018 to 2023

**DOI:** 10.3389/fmed.2024.1390920

**Published:** 2024-04-29

**Authors:** Wanli Zang, Jin Yan

**Affiliations:** ^1^Postgraduate School, Harbin Sport University, Harbin, China; ^2^School of Physical Education and Sports Science, Soochow University, Suzhou, China

**Keywords:** lower back pain, exercise, bibliometric analysis, CiteSpace, global research

## Abstract

**Objective:**

This study aims to explore global research trends on exercise interventions for nonspecific low back pain from 2018 to 2023 through bibliometric analysis.

**Methods:**

A systematic search was conducted in the Web of Science Core Collection database to select relevant research articles published between 2018 and 2023. Using CiteSpace and VOSviewer, the relationships and impacts among publications, different countries, journals, author groups, references, and keywords were analyzed in depth.

**Results:**

The bibliometric analysis included 4,896 publications, showing a trend of initial growth followed by a decline. At the national level, the United States made the most significant contributions in this field. The journal “Lancet” had three of the top 10 most-cited articles, with an average citation count of 306.33, and an impact factor reaching 168.9 in 2023. The analysis also revealed that “disability,” “prevalence,” and “management” were high-frequency keywords beyond the search terms, while “rehabilitation medicine,” “experiences,” and “brain” emerged as new hotspots in the research.

**Conclusion:**

This study reveals the global trends in research on exercise interventions for nonspecific low back pain over the past 5 years and highlights potential research frontiers in the field. These findings provide a solid foundation for focusing on key issues, potential collaboration directions, and trends in research development in the future, offering valuable references for further in-depth studies.

## 1 Introduction

Nonspecific low back pain (NSLBP) refers to pain in any region of the back between the rib margin and the gluteal folds, often accompanied by symptoms of lower limb discomfort, representing a syndrome characterized by back pain (1, 2). It’s a common clinical complaint and symptom, arising from various known or unknown abnormalities or diseases. The global prevalence of LBP is as high as 7.3% (3). Between 2004 and 2012, the global incidence of LBP increased by 2.9% (4), while disability due to back pain surged by 54% from 1990 to 2015 (5). A survey encompassing nearly 200,000 individuals across 43 countries revealed that individuals with LBP are twice as likely to suffer from depression, anxiety, psychiatric disorders, or sleep deprivation compared to those without LBP (6). The lifetime prevalence of LBP is between 70 and 80%, with a significant portion of patients (10 to 20%) developing chronic LBP lasting at least 3 months (7). Although traditional medical approaches like medication and surgery play a crucial role in treating LBP, they often come with potential side effects and high costs (8). In light of this, exercise therapy, as a non-pharmacological intervention, is increasingly favored by physicians and patients alike, owing to its minimal side effects, low cost, and ease of implementation.

In addressing the widespread and challenging health issue of lower back pain, exercise intervention strategies have demonstrated significant therapeutic potential. The mechanisms of action of exercise interventions can be analyzed across multiple physiological dimensions (9–11). Regular exercise, particularly targeted training of core muscle groups such as the abdominal and back muscles, significantly strengthens spinal support, effectively reducing the burden on the lower back (12). Physical activity enhances the flexibility of the spine and its surrounding tissues, aiding in the alleviation of muscle stiffness and discomfort (13). Moderate aerobic exercise promotes blood circulation and improves tissue nutrition, thereby contributing to the reduction of inflammation and facilitating the self-repair processes of tissues (14). Furthermore, physical activities have a role in modulating the function of the nervous system, potentially reducing neural sensitivity in cases of chronic pain (14).

Although exercise interventions are widely recognized as effective treatments for lower back pain, research in this area remains relatively fragmented globally. Current studies on exercise therapy for lower back pain, particularly those providing a comprehensive global analysis, are crucial for clinical practitioners and researchers. This study plans to conduct a visual analysis of the literature related to the treatment of NSLBP from August 1, 2018, to August 1, 2023, using CiteSpace and VOSviewer tools, based on the Web of Science database. The aim is to reveal the knowledge structure, primary research forces, keyword co-occurrence networks, as well as research hotspots and trends in the field of NSLBP treatment, especially in the area of exercise interventions. This analysis will not only enable professionals in the field to gain a more comprehensive understanding of the latest advancements in the treatment of NSLBP but also provide direction for future research and establish a more solid scientific foundation for clinical applications.

## 2 Methods

### 2.1 Search strategy and data acquisition

This study selected the Web of Science (WoS) as its primary data source, a decision grounded in WoS’s unique strengths in providing detailed citation information, author, country, and journal data. Furthermore, considering the differences in search strategies and result interpretation across various databases, utilizing WoS helps mitigate potential biases, ensuring that our analysis results maintain a high level of accuracy and reliability. This article, as of August 1, 2023, with the following search strategy: #1: Topic includes “Low Back Pain” OR “Lumbago” OR “Lumbar Pain” OR “Backache” OR “Non-specific Low Back Pain” OR “Non-specific Lumbago” OR “Non-specific Back Pain” OR “Chronic Low Back Pain” OR “Acute Low Back Pain” OR “Mechanical Low Back Pain” OR “Idiopathic Low Back Pain” OR “Simple Low Back Pain”; #2: Topic includes “Spinal Stenosis” OR “Lumbar Stenosis” OR “Intervertebral Disc Displacement” OR “Disc Herniation” OR “Disc Protrusion” OR “Sciatica” OR “Lumbar Radiculopathy” OR “Spondylolisthesis” OR “Lumbar Disc Disease” OR “Ankylosing Spondylitis” OR “Lumbar Spondylitis” OR “Lumbar Spondylosis” OR “Lumbar Osteoarthritis” OR “Spinal Tumors” OR “Metastatic Spinal Tumors” OR “Spinal Infections” OR “Vertebral Osteomyelitis” OR “Lumbar Fractures” OR “Vertebral Fractures” OR “Lumbar Vertebral Fractures” OR “Compression Fractures” OR “Kidney Stones” OR “Urinary Tract Infections” OR “Lumbar Strain” OR “Lumbar Sprain” OR “Lumbar Muscle Strain” OR “Lumbar Muscle Sprain”; #3: Topic includes “Exercise” OR “Physical Activity” OR “Physical Exercise” OR “Acute Exercise” OR “Aerobic Exercise” OR “Anaerobic Exercise” OR “Exercise Training” OR “Strength Training” OR “Weight Lifting” OR “Weight-bearing Exercise” OR “Resistance Training” OR “High Intensity Interval Training” OR “Yoga” OR “Tai Chi” OR “Pilates” OR “Dance” OR “Walking” OR “Running” OR “Cycling” OR “Swimming” OR “Sports” OR “Fitness” OR “Workout” OR “Gym” OR “Training” OR “Jogging” OR “Hiking” OR “Treadmill” OR “Elliptical” OR “Stationary Bike” OR “Rowing Machine” OR “Stair Climber” OR “Dumbbell” OR “Barbell” OR “Resistance Bands” OR “Kettlebell.” The final search strategy was #1 not #2 and #3. The document type was limited to “Article or Review,” with publication dates ranging from August 1, 2018, to August 1, 2023, yielding a total of 4,896 publications related to the impact of exercise interventions on non-specific pain. The PubMed IDs (PMID numbers) of the retrieved documents were used to obtain citation data from the WoS Core Collection, and the ‘full record and cited references’ were imported in txt format into VOSviewer 1.6.18.0 (15) and CiteSpace 5.1.R8 SE (32-bit) (16) for analysis.

### 2.2 Software and data analysis

Microsoft Excel 2016 software was utilized to analyze information related to exercise interventions for non-specific pain, encompassing aspects such as the year of publication, journals, authors, institutions, countries/regions, citations, and keywords. Bibliometric analysis software VOSviewer 1.6.18.0 and CiteSpace 5.1.R8 SE (32-bit) were employed to systematically analyze the distribution and collaboration among authors, institutions, and countries, as well as co-citations and keywords, subsequently facilitating the creation of visual mapping representations.

## 3 Results

### 3.1 Publication volume and trend analysis

**Figure 1:**
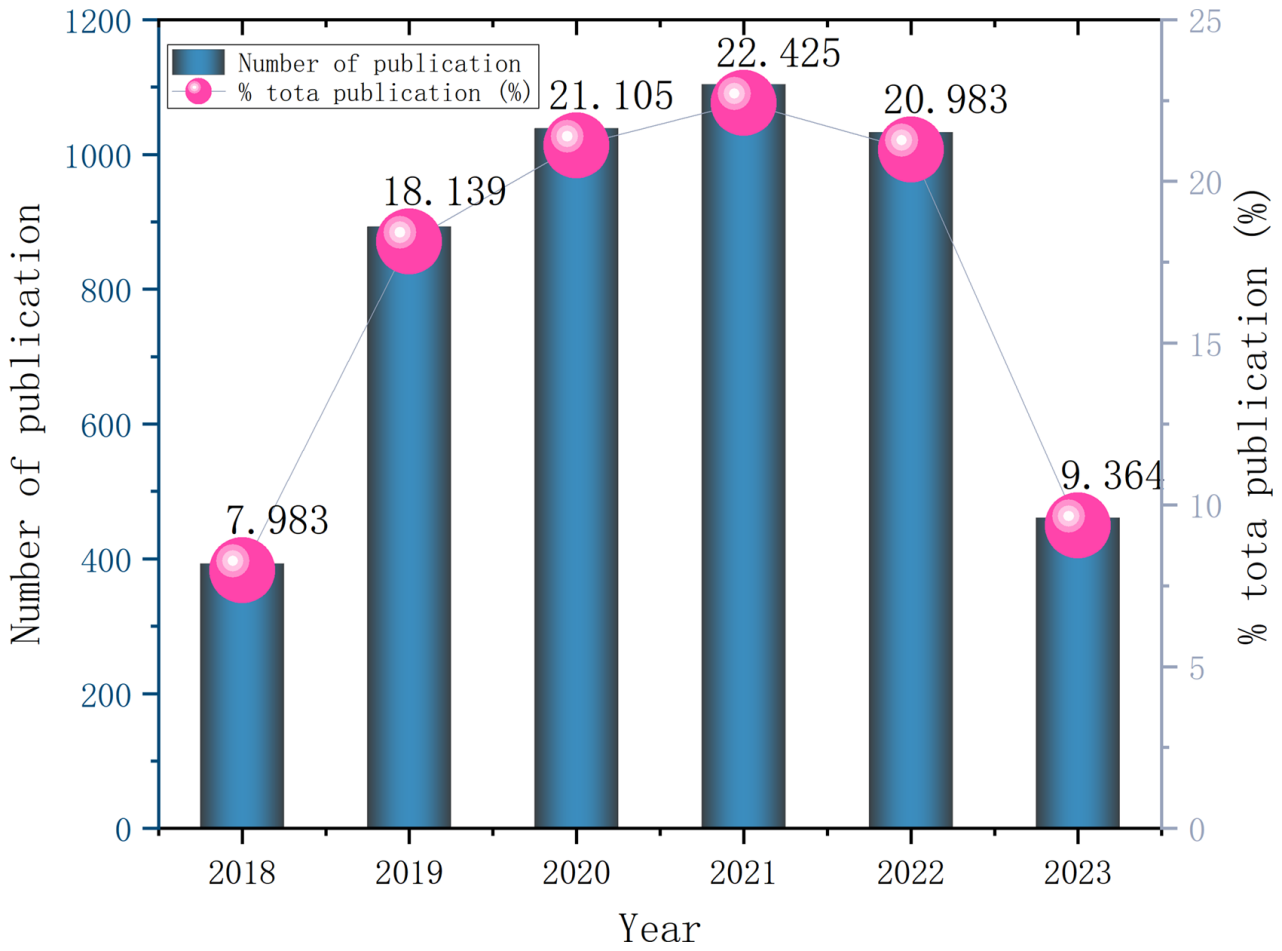
Trend of publication volume from 2018 to 2023.

This study incorporated a total of 4,896 articles. An analysis of the publication years of these 4,896 documents revealed an overall upward trend in the number of papers related to the impact of exercise interventions on non-specific pain. Notably, the peak was reached in 2021, with a total of 1,104 publications, representing a significant increase of 22,425% from the previous year (Figure 1).

### 3.2 Analysis of international collaboration

**Figure 2:**
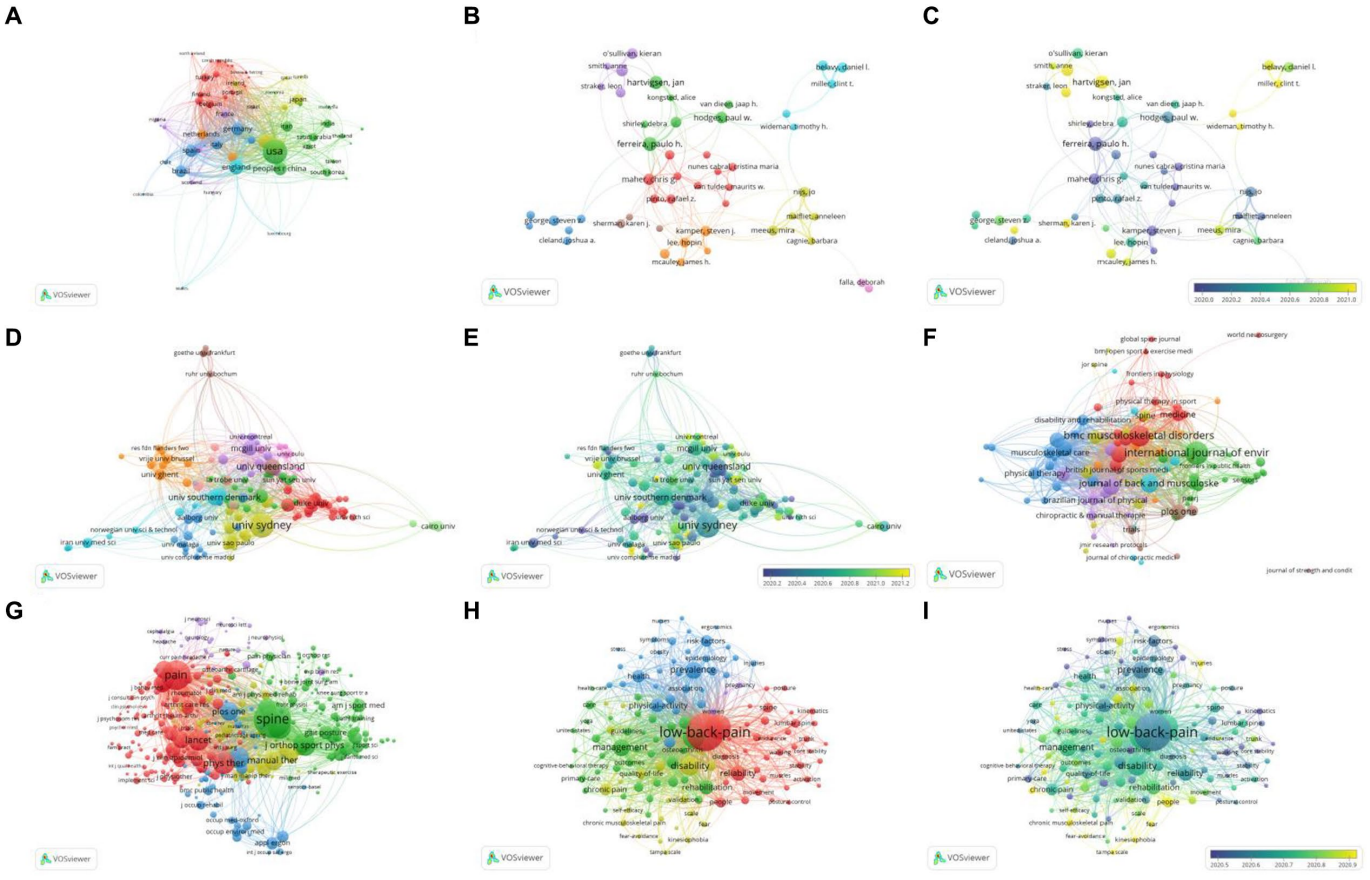
Figure created using VOSviewer. **(A)** Visualization of countries/regions. **(B)** Visualization map of authors. **(C)** Visualization map of average publication year. **(D)** Visualization map of research institutions. **(E)** Visualization map of average publication year by institution. **(F)** Visualization map of cited journals. **(G)** Visualization map of co-cited journals. **(H)** Visualization map of keywords. **(I)** Visualization map of keywords.

Between 2018 and 2023, a total of 115 countries/regions participated in research related to the impact of exercise interventions on non-specific pain. Table 1 lists the top 10 countries/regions by publication volume. The United States emerged as the leading country in terms of publication volume in this field (*n* = 1,160), accounting for over 23% of all papers, followed by Australia (*n* = 550) and the United Kingdom (*n* = 408). Notably, despite the United States having the highest publication volume, its average citation index (ACI) was not as high as that of Australia. Visual analysis revealed the overall strength of the linkages between countries as follows: Australia (TLS = 700), the United States (TLS = 688), and the United Kingdom (TLS = 576). Furthermore, extensive international collaborations were observed, particularly among the United States, the United Kingdom, Spain, Brazil, Germany, and Iran (Figure 2A).

**Table 1:** Top 10 countries/regions by publication volume.

Rank	Country/region	Number of publications	Citation frequency	Average citation index (ACI)	Percentage of total publications (%)	Total link strength
1	United States	1,160	9,662	8.33	23.563	688
2	Australia	550	6,794	12.35	11.172	700
3	United Kingdom	408	3,993	9.79	8.288	576
4	Canada	379	4,154	10.96	7.699	476
5	China	379	2,540	6.70	6.927	155
6	Germany	318	2,900	9.12	6.459	363
7	Spain	286	2,153	7.53	5.809	257
8	Brazil	271	2,626	9.69	5.505	192
9	Japan	230	912	3.97	4.672	54
10	Iran	205	1,235	6.02	4.164	101

### 3.3 Analysis of the authors’ collaboration network

A total of 19,611 authors contributed to the research on the impact of exercise interventions on non-specific pain. Table 2 summarizes the top 10 authors by publication volume, with Ferreira P. H. (37 papers), Maher C. G. (36 papers), and Hartvigsen J. (33 papers) being the most prolific. The international collaboration among authors is visually depicted in Figure 2B. Each node on the map represents an author, with the size of the circle reflecting the number of publications by that author, and the lines connecting the circles indicating co-authorship relationships. Furthermore, a superimposed visual map (Figure 2C) intuitively displays the average publication year of the authors, with authors in the green cluster identified as pioneers in the field of exercise intervention for non-specific pain, whereas those in the yellow cluster are authors who have published more recently.

**Table 2:** Top 10 authors by publication volume.

Rank	Author	Number of publications	Citation frequency	Average citation frequency
1	Ferreira P. H.	37	915	24.73
2	Maher C. G.	36	1,747	48.53
3	Hartvigsen J.	33	216	6.55
4	Hodges P. W.	26	438	16.85
5	Ferreira M. L.	25	141	5.64
6	O’Sullivan P.	24	391	16.29
7	Lee H.	23	104	4.52
8	Pinto R. Z.	21	666	31.71
9	Andersen L. L.	20	97	4.85
10	Hancock M. J.	20	107	5.35

### 3.4 Analysis of institutional collaboration

The number of publications by a scientific institution is indicative of its research capacity in a particular field. Among the 4,896 documents analyzed, there were contributions from 5,799 research institutions. Figure 2D displays the network of 136 institutions with international collaborations that have published more than 15 papers, illustrating the close cooperation between these entities. The top 10 institutions contributed 903 papers, accounting for 18.344% of all indexed articles (Table 3). As indicated in Table 3, the top three institutions in terms of publication volume are the University of Sydney (*n* = 178), Harvard University (*n* = 98), and the University of Queensland (*n* = 93). By superimposing publication times on the network of institutional collaboration, a temporal overlay map of institutions was generated, with different colors representing the average year of appearance in the literature. This approach revealed the evolutionary trend of research institutions in the field. Institutions such as Karolinska Institutet, Universidade Federal de Minas Gerais, University of Oulu, University of Sevilla, and Chiropractic Knowledge Hub, indicated in yellow in the cluster, emerged as the latest research hotspots in the field of exercise interventions for non-specific pain (Figure 2E).

**Table 3:** Top 10 institutions by publication volume.

Rank	Institution		Number of publications	Citation frequency	Percentage of total publications (%)
1	University of Sydney	Australia	178	3,092	3.616
2	Harvard University	United States	98	1,124	1.991
3	University of Queensland	Australia	93	1,100	1.889
4	University of Southern Denmark	Denmark	90	1,461	1.828
5	University of California System	United States	89	1,096	1.808
6	Vrije Universiteit Amsterdam	Netherlands	84	1,594	1.706
7	McGill University	Canada	72	628	1.463
8	Harvard Medical School	United States	69	803	1.402
9	Curtin University	Australia	66	1,055	1.341
10	University of London	United Kingdom	64	319	1.300

### 3.5 Analysis of relevant journals

**Figure 3:**
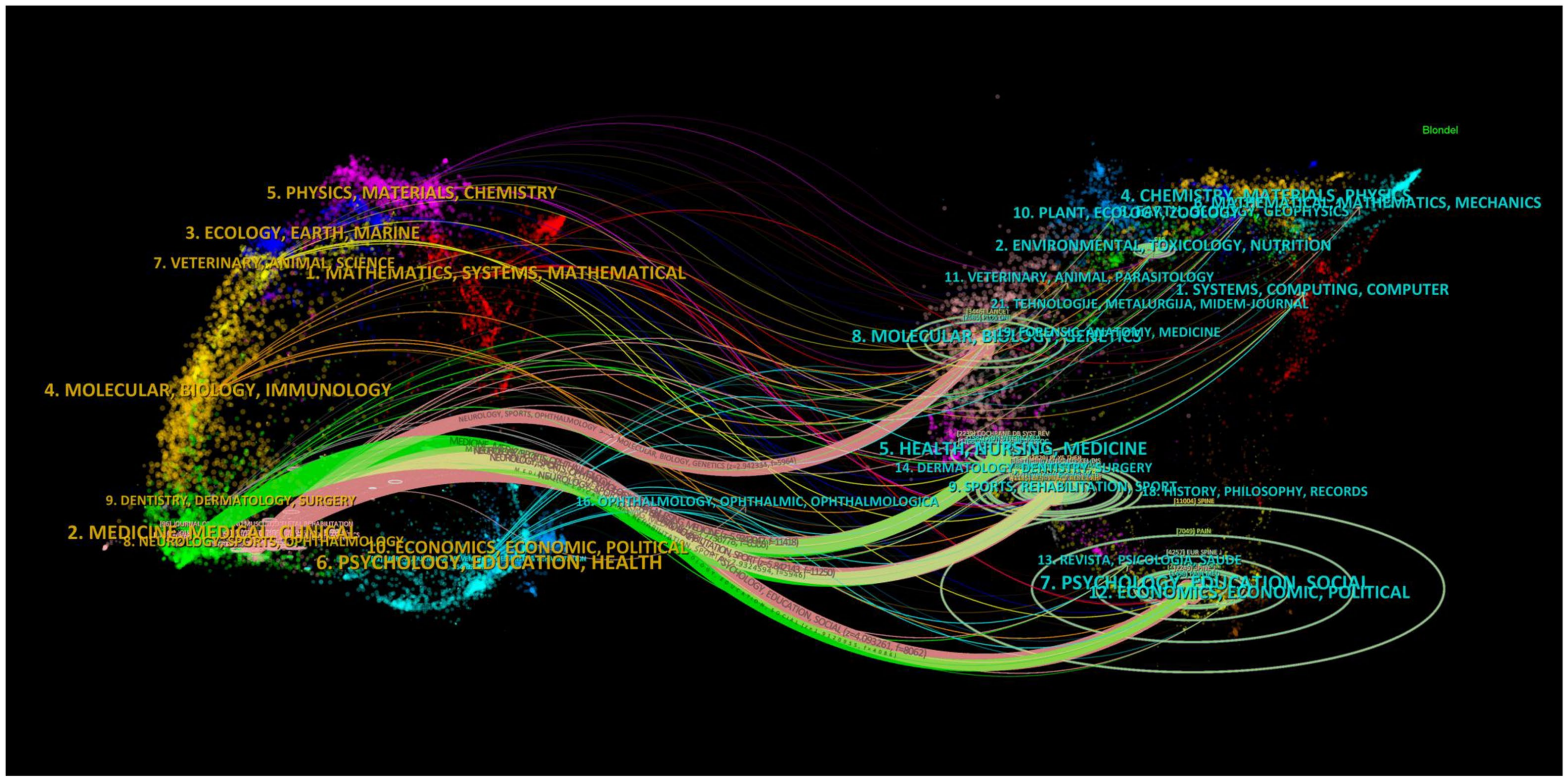
Dual-overlay map of journals.

This study conducted a comprehensive analysis of the contributions of journals in the field of exercise interventions for non-specific pain, with articles published across 1,125 journals. Table 4 lists the 10 most influential journals in this research domain. Notably, the International Journal of Environmental Research and Public Health (*n* = 147), BMC Musculoskeletal Disorders (*n* = 125), and Journal of Back and Musculoskeletal Rehabilitation (*n* = 96) rank as the top three journals in terms of publication volume. Among the 10 most productive journals, the International Journal of Environmental Research and Public Health boasts the highest Impact Factor (4.614) and a total citation count of 1,034. VOSviewer was used to create visual maps of cited and co-cited journals, as shown in Figures 2F,G, revealing that many journals appear in both maps and have close citation relationships. Additionally, a dual-overlay map of the journals depicts the distribution of themes within the journals (Figure 3). Citing and cited journals are positioned on the left and right sides of the image, respectively, with colored pathways indicating citation relationships. It is evident that there are three main pathways between citing and cited journals, with the strongest citation relationship being from Medicine/Medical/Clinical journals to Psychology/Education/Social journals.

**Table 4:** Top 10 journals by publication volume.

Rank	Journal	Number of publications	Total citation frequency	Impact factor (2022)	Journal citation reports
1	International Journal of Environmental Research and Public Health	147	1,034	4.614	Q1
2	BMC Musculoskeletal Disorders	125	828	2.3	Q3
3	Journal of Back and Musculoskeletal Rehabilitation	96	270	1.6	Q4
4	BMJ Open	88	379	2.9	Q3
5	Musculoskeletal Science and Practice	75	487	2.3	Q2
6	PLoS One	73	469	3.7	Q3
7	Physiotherapy Theory and Practice	71	367	2	Q4
8	Medicine	55	308	1.6	Q4
9	Pain Medicine	55	404	3.1	Q3
10	Journal of Pain Research	50	357	2.7	Q3

### 3.6 Analysis of co-cited references

**Figure 4:**
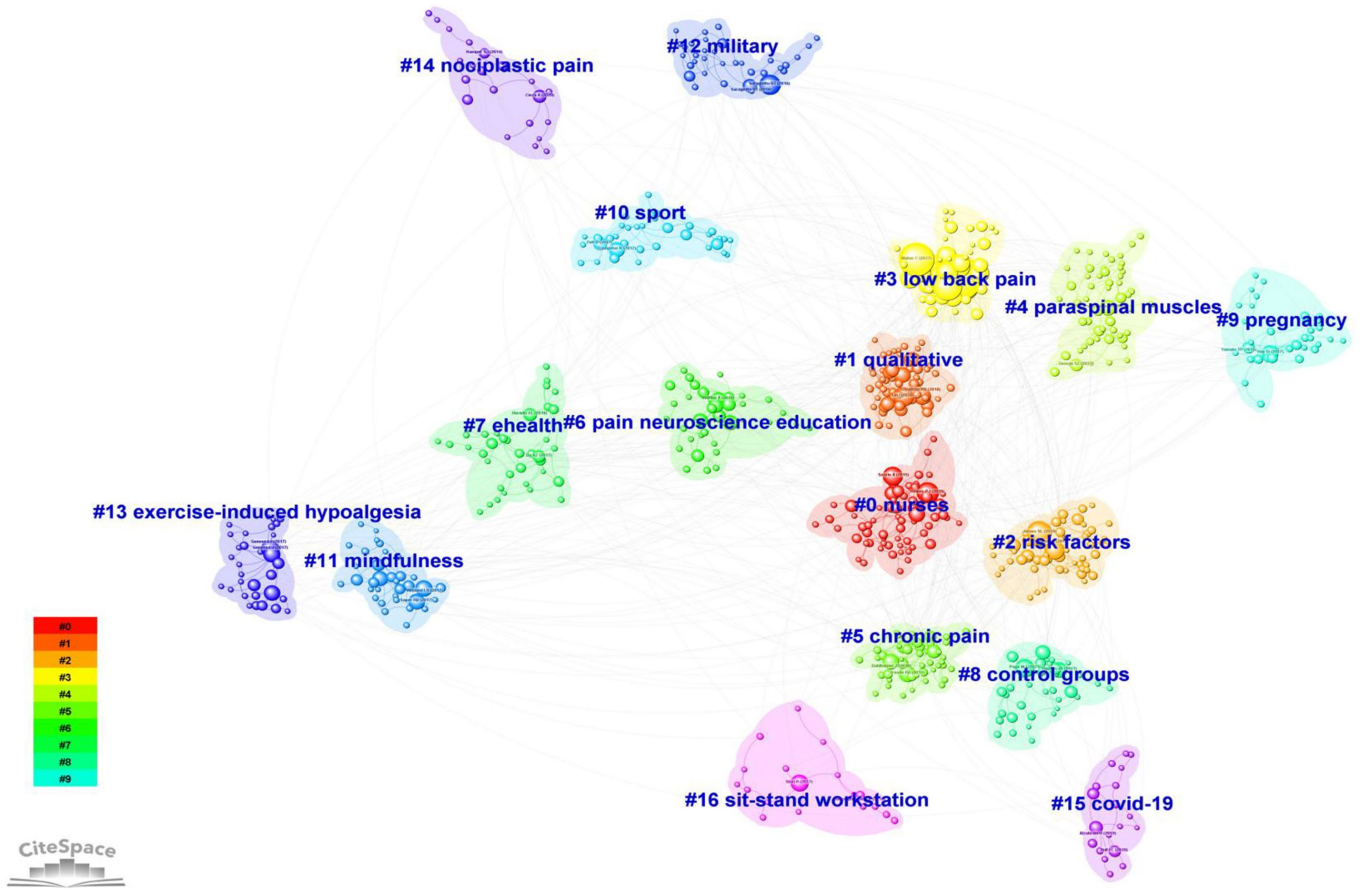
Cluster analysis map of co-cited references.

**Figure 5:**
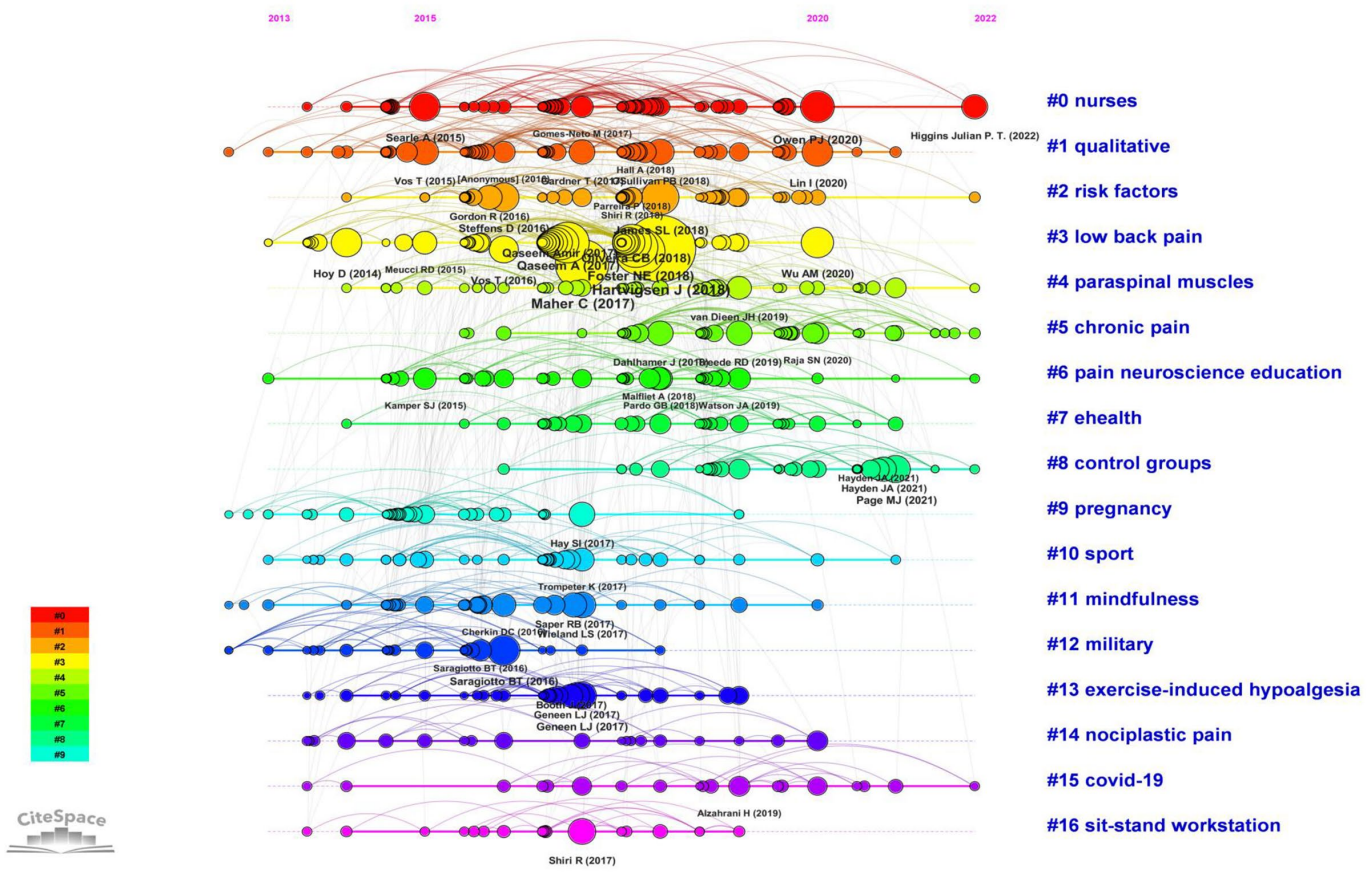
Timeline view of co-cited references.

**Figure 6:**
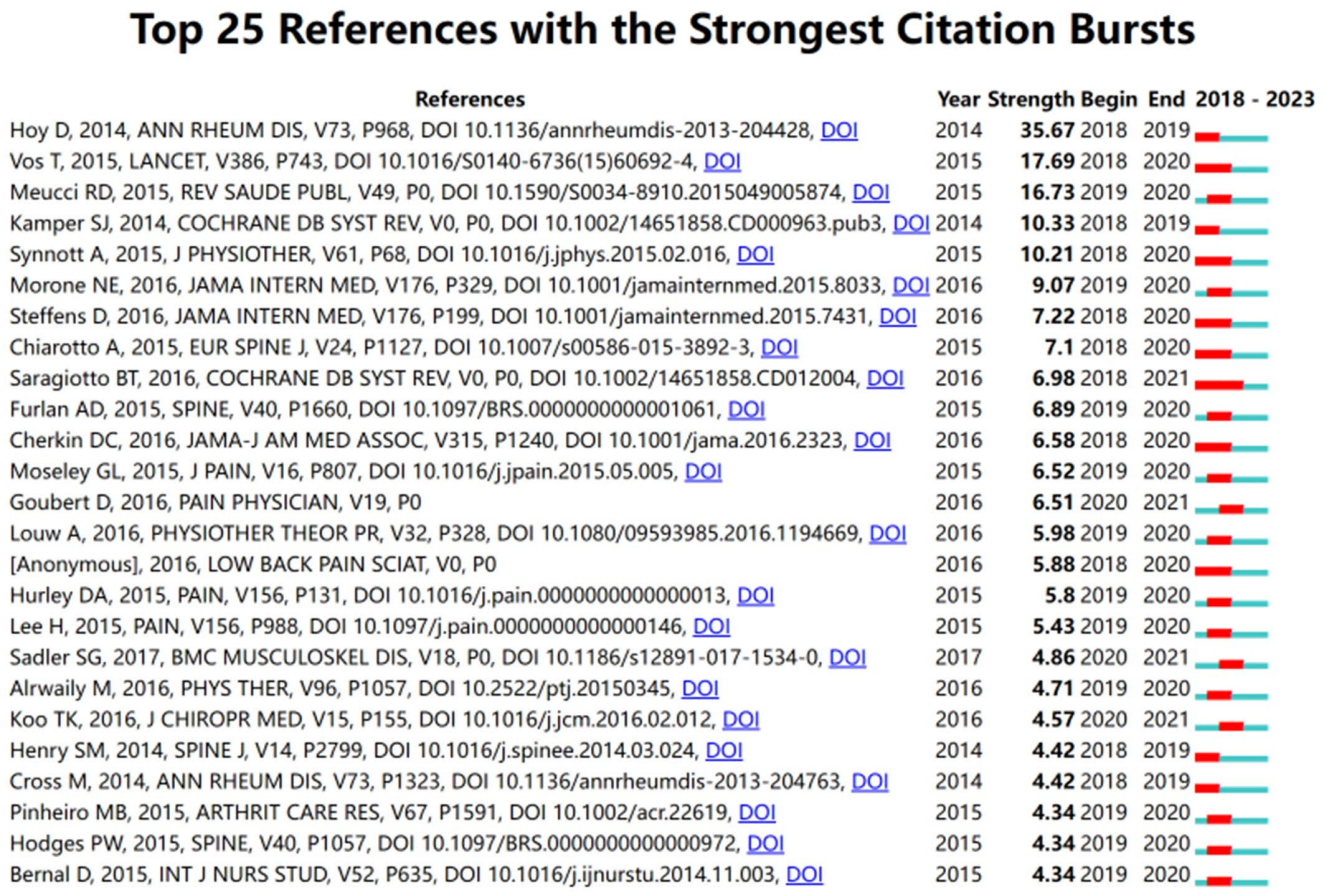
Burst detection map of co-cited references.

Table 5 lists the top 10 most frequently cited references in the field of exercise interventions for non-specific pain. The most cited article is by Hartvigsen et al. (3), published in The Lancet, with 444 citations, followed by Maher et al. (1) in The Lancet, cited 285 times, and Hoy et al. (17) in Ann Rheum Dis, with 262 citations. A visual analysis of co-cited references was conducted using CiteSpace (Figure 4), which also clustered the co-cited references, revealing 17 clusters. The first cluster is labeled “#0 nurses,” the second as “#1 qualitative,” the third as “#2 risk factors,” the fourth as “#3 low back pain,” and the fifth as “#4 paraspinal muscles.” Additionally, a timeline view was used to display the evolution of co-cited references over time (Figure 5). This timeline view, a data visualization technique combining clustering and time-slicing, sorts cluster labels based on whether they appeared earlier or later after clustering. This not only illustrates the distribution of themes in the field but also shows the trends and interrelationships of research topics over time. In the timeline view, nodes in the same row with different colors represent different years, with left-side nodes indicating older keywords and right-side ones representing recent keywords. Lines on the same horizontal level represent the collective citations of all clusters in that row, with cluster labels at the right end of the line. As shown in Figure 5, “#1 qualitative,” “#9 pregnancy,” “#11 mindfulness,” and “#12 military” represent early research directions in this field. Furthermore, clusters such as “#5 chronic pain” and “#8 control groups” at the right end of the timeline view highlight current research focuses in the field. As depicted in Figure 6, prominent co-cited references in this area include works by Goubert D. (2016), Sadler S. G. (2017), and Koo T. K. (2016).

**Table 5:** Top 10 journals by citation count.

Title	Journal	First author	Year	Total citation frequency
What low back pain is and why we need to pay attention	Lancet	Jan Hartvigsen	2018	444
Non-specific low back pain	Lancet	Chris Maher	2017	285
The global burden of low back pain: estimates from the Global Burden of Disease 2010 study	Ann Rheum Dis	Damian Hoy	2014	262
A systematic review of the global prevalence of low back pain	Arthritis Rheum	Damian Hoy	2012	253
The Oswestry Disability Index	Spine	J. C Fairbank	2000	253
Chapter 4. European guidelines for the management of chronic nonspecific low back pain	Eur Spine J	O. Airaksinen	2006	212
A Fear-Avoidance Beliefs Questionnaire (FABQ) and the role of fear-avoidance beliefs in chronic low back pain and disability	Pain	Gordon Waddell	1993	209
Non-specific low back pain	Lancet	Federico Balagué	2012	190
Noninvasive treatments for acute, subacute, and chronic low back pain: a clinical practice guideline from the American College of Physicians	Ann Intern Med	Amir Qaseem	2017	184
Exercise interventions for the treatment of chronic low back pain: a systematic review and meta-analysis of randomised controlled trials	Clin Rehabil	Angela Searle	2015	168

### 3.7 Keyword analysis

**Figure 7:**
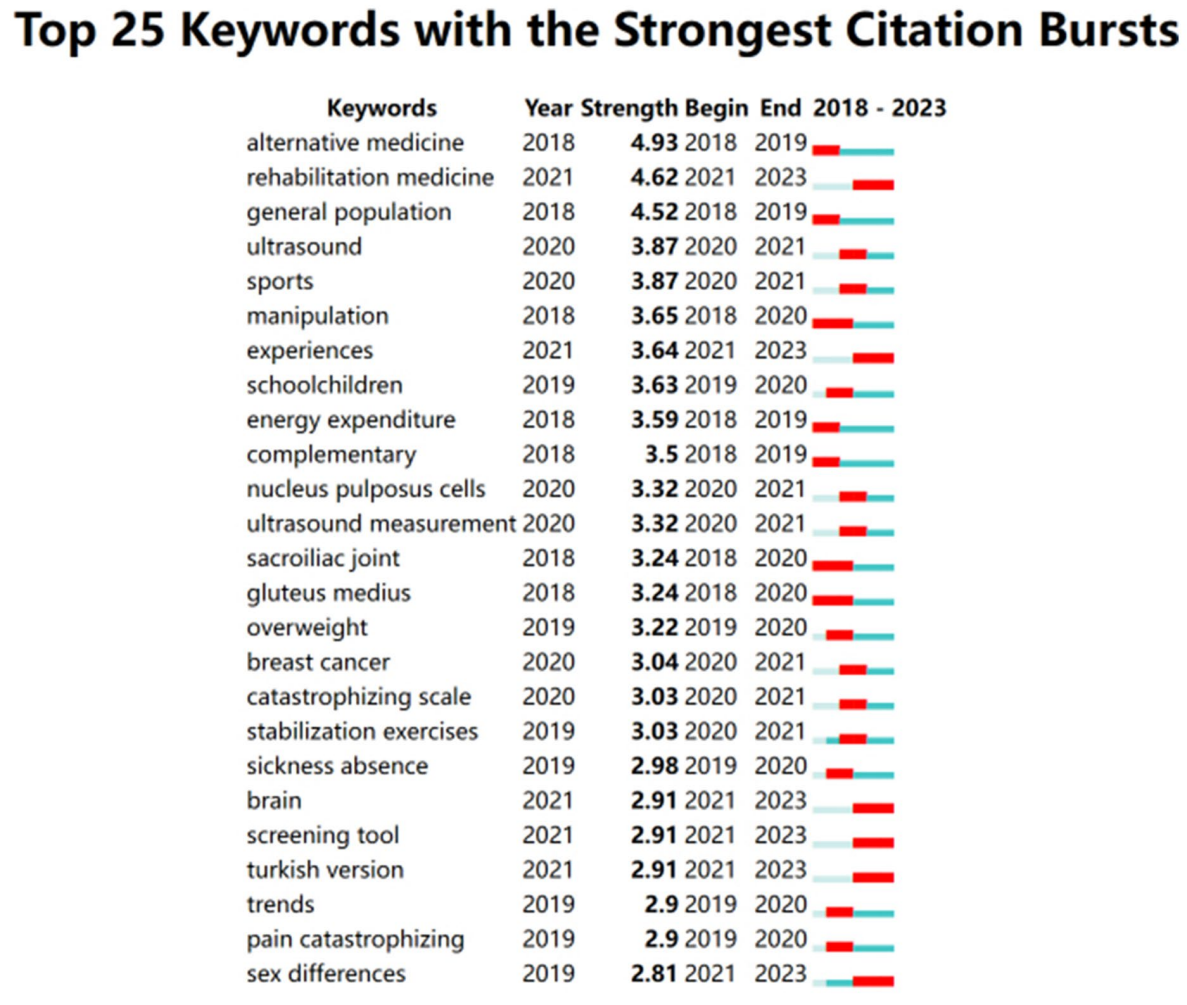
Burst detection map of co-cited keywords.

In the 4,896 articles analyzed, a total of 12,815 keywords were identified, among which 135 keywords appeared at least 60 times (Figure 2H). The accuracy and frequency of keywords are two crucial factors affecting the precision of co-occurrence methods in identifying research hotspots within a field. To enhance the accuracy of the analysis, an initial organization of the keywords in the literature was conducted, which included unifying the capitalization, singular/plural forms, acronyms/abbreviations, and synonyms. Table 6 lists the top 10 most frequently occurring keywords in the field of exercise interventions for non-specific pain, with “low-back-pain” being the most common (2,264 occurrences), followed by “low back pain” (1,117 times), and “exercise” (895 times). As indicated in Figure 2I, keywords such as “people,” “fear-avoidance,” “quality,” “injuries,” “chronic pain,” and “chronic musculoskeletal pain” are indicative of recent research trends. Figure 7 shows that “brain” (2021), “screening tool” (2021), “Turkish version” (2021), and “sex differences” (2019) are currently prominent cited keywords in this field.

**Table 6:** Top 10 keywords.

Rank	Keyword	Frequency
1	Low-back-pain	2,264
2	Low back pain	1,117
3	Exercise	895
4	Disability	727
5	Prevalence	605
6	Management	603
7	Reliability	482
8	Rehabilitation	438
9	Physical-activity	418
10	Health	369

## 4 Discussion

### 4.1 Research trends in exercise interventions for NSLBP

To our knowledge, this is the first bibliometric analysis of exercise interventions for NSLBP based on the Web of Science Core Collection. Utilizing the CiteSpace tool, we analyzed 4,896 papers published in this field from 2018 to 2023, revealing ongoing academic efforts in this domain. Results indicate a steady annual increase in the number of publications. However, the rapid growth in publication volume does not necessarily equate to high quality; it merely suggests a growing interest among researchers in exercise interventions for lower back pain. The top 10 journals in this field do not have particularly high impact factors, with only one within the top 25% and the remaining nine falling in the lower 50%. Moreover, significant disparities are observed among different countries, researchers, and academic institutions. Globally, countries such as the United States, Australia, the United Kingdom, Canada, and China are at the forefront of research activities in this field. This may reflect these nations’ advantages in medical research infrastructure and funding, as well as their emphasis on improving public health quality (18). Regarding author influence, scholars such as Ferreira P. H., Maher C. G., and Hartvigsen J. have played pivotal roles in advancing the field. Their work has enriched our theoretical understanding of NSLBP treatment and offered significant practical guidance. Institutionally, the University of Sydney in Australia, Harvard University in the United States, and the University of Southern Denmark are leading in publication volume in the field of exercise intervention for lower back pain. The exceptional depth and breadth of research by these institutions mark their academic leadership on a global scale. Notably, the geographical distribution and concentration of academic resources among these institutions highlight the uneven distribution of global research forces.

### 4.2 Research focus in exercise interventions for NSLBP

Based on co-occurrence network analysis of keywords, excluding the search terms, the most frequent keywords were “disability,” “prevalence,” and “management,” indicating that the current research focus in the domain of exercise interventions for NSLBP revolves around the management of disability, prevalence of the condition, and its management strategies. The top emerging keywords were “rehabilitation medicine,” “experiences,” and “brain,” suggesting potential new trends. These three research trends are as follows:

1. Rehabilitation Medicine: Research in the field of rehabilitation medicine on exercise interventions for NSLBP shows positive progress. Modified exercise therapies incorporating relaxation techniques significantly reduce the intensity of pain and anxiety in office workers with chronic lower back pain, improving their range of motion (19). Additionally, physical exercises at the workplace enhance symptoms, flexibility, range of motion, muscle strength, and quality of life in office workers with lower back pain (20). These studies underscore the importance of exercise interventions in rehabilitation medicine for the treatment of lower back pain, offering not just pain relief but also improved functionality and quality of life. These findings support the critical role of exercise interventions in rehabilitation medicine, particularly in managing chronic lower back pain.
2. Experiences: Participation in exercise programs not only alleviates physical symptoms but also enhances psychological well-being and self-efficacy among patients (21). Furthermore, engagement in such programs increases patients’ confidence in and compliance with treatment, demonstrating positive psychological effects (22). However, it is important to note that sustained participation and adaptability to exercise remain challenging for some patients (22). This indicates that while exercise interventions hold potential in increasing patient acceptance of treatment, individual differences and challenges in sustained participation need to be considered in implementing these interventions.
3. Brain: Exercise training targeted at NSLBP may improve cognitive performance and reduce functional connectivity in brain regions associated with pain (23). Active physical activity can act as a protective factor against NSLBP and psychological health complications (24). These studies emphasize the importance of exercise interventions in rehabilitation medicine for treating lower back pain, particularly regarding their impact on brain function and pain processing. Through these interventions, patients may not only achieve significant progress in pain relief and functional improvement but also experience positive changes in brain function and neurobiology.

From the top five most cited references, we can identify potentially useful references for exploring the knowledge frontier of research. NSLBP is associated with physical, psychological, and social factors such as manual labor, obesity, smoking, mental stress, and low socioeconomic status. While most new cases of NSLBP recover quickly, there is a high recurrence rate, with a minority developing into persistent and disabling conditions. Therefore, attention to NSLBP is needed, particularly in exercise and daily activities, to prevent and manage this issue (3). Management includes education and reassurance, analgesic medications, non-pharmacological therapies, and timely follow-up. There are two treatment strategies: a stepped approach starting with simpler care and progressing to more intensive care if the patient does not respond, and the use of simple risk prediction methods to personalize the amount and type of care provided. Moreover, the overuse of imaging, opioids, and surgery remains a widespread issue (3). NSLBP is the most disabling of all 291 conditions and ranks sixth in overall burden. Analysis of the prevalence, incidence, remission, duration, and risk of death from NSLBP reveals that the prevalence and burden of NSLBP increase with age (17). Researchers are advised to use the recently recommended standard definitions of NSLBP and refer to the recently developed tools for assessing bias risk in prevalence studies (4). The Oswestry Disability Index measures how NSLBP affects a patient’s daily activities and functionality. It includes 10 questions about pain intensity, personal care, lifting, walking, sitting, sleeping, social life, traveling, and employment. The total score ranges from 0 to 50, with higher scores indicating greater disability (25). Concurrently, scientific research has unveiled a direct link between patient expectations and therapeutic outcomes, underscoring the imperative to delve deeper into this subject. The research conducted by Ballestra et al. (26) highlights the significant impact of patients’ strong anticipation for personalized training and regular follow-up, as well as their optimistic hopes for treatment results. Whether it is an active pursuit of pain relief and enhanced activity levels or a realistic or resigned attitude towards these improvements, such expectations considerably influence recovery efficacy. Moreover, effective communication, the fulfillment of needs as recognized individuals, and a clear explanation of the pain’s origins are equally associated with superior recovery outcomes.

### 4.3 Clinical implications

The bibliometric analysis of exercise interventions for NSLBP spanning 2018 to 2023 accentuates the critical importance of tailoring exercise regimens to individual patient needs, a cornerstone of patient-centered care. This research highlights the imperative for clinicians to adopt customized treatment plans that are informed by a detailed understanding of the patient’s unique health profile, integrating these into everyday clinical practice. It underscores the necessity for policy interventions that support the deployment of such personalized care strategies, aiming to mitigate the disparities in research and the accessibility of care across different regions. Future research endeavors should focus on deciphering the mechanisms that underpin the efficacy of personalized exercise interventions, assessing their long-term benefits, and incorporating patient feedback to refine NSLBP management approaches. Embracing such methodologies promises not only to reduce physical discomfort but also to elevate mental health and overall life quality, advocating for a comprehensive NSLBP care strategy that emphasizes patient education and active participation. This shift towards more refined, patient-specific care models requires a collaborative effort among clinicians, researchers, and policymakers alike to democratize access to high-quality NSLBP care and equalize research opportunities on a global scale.

### 4.4 Strengths and limitations

This study represents the first comprehensive bibliometric analysis of the advancements and trends in the field of exercise intervention for NSLBP over the past 5 years, based on data from the Web of Science. To ensure the comprehensiveness and diversity of data, this analysis included 4,896 publications. Moreover, this bibliometric analysis not only encompassed publications, journals, citation counts, authors, and the collaboration network of countries and regions, but also meticulously examined emerging keywords, thematic research keywords, keyword co-occurrence networks, and the top 10 most cited references.

However, the study also has some limitations. Firstly, as the study only included English-language papers, excluding literature in other languages, this may introduce publication bias. Secondly, despite analyzing collaboration networks, the study did not delve into specific connections among authors, regions, or countries.

## 5 Conclusion

This study, through an analysis of research trends in exercise interventions for NSLBP over the past 5 years, has revealed potential research frontiers in the field, providing valuable insights for the development of more effective exercise intervention measures for lower back pain. The interest in exercise intervention as a treatment method for NSLBP has been continuously rising among clinicians and researchers. The findings of this analysis not only lay the groundwork for collaboration among research teams but also hold the promise of promoting wider clinical application of exercise therapy in the management of lower back pain. Although this study has its limitations, it provides a solid foundation for in-depth exploration of the popular topic of exercise treatment for lower back pain. It also establishes an important cornerstone for identifying research priorities, building partnerships, and predicting future trend.

## Data availability statement

The original contributions presented in the study are included in the article/supplementary material, further inquiries can be directed to the corresponding author.

## Author contributions

WZ: Data curation, Methodology, Supervision, Writing – original draft, Writing – review & editing. JY: Writing – original draft, Writing – review & editing.
